# New Window Into Hepatitis B in Africa: Liver Sampling Combined With Single-Cell Omics Enables Deep and Longitudinal Assessment of Intrahepatic Immunity in Zambia

**DOI:** 10.1093/infdis/jiae054

**Published:** 2024-02-09

**Authors:** Taonga Musonda, Michael S Wallace, Hailey Patel, Owen P Martin, Christopher Oetheimer, Simutanyi Mwakamui, Edford Sinkala, Bright Nsokolo, Annie Kanunga, Georg Lauer, Raymond T Chung, Gilles Wandeler, Debika Bhattacharya, Paul Kelly, Nadia Alatrakchi, Michael J Vinikoor

**Affiliations:** Tropical Gastroenterology and Nutrition Group, University of Zambia, Lusaka, Zambia; Division of Gastroenterology, Massachusetts General Hospital, Boston, Massachusetts, USA; Division of Gastroenterology, Massachusetts General Hospital, Boston, Massachusetts, USA; Division of Gastroenterology, Massachusetts General Hospital, Boston, Massachusetts, USA; Division of Gastroenterology, Massachusetts General Hospital, Boston, Massachusetts, USA; Tropical Gastroenterology and Nutrition Group, University of Zambia, Lusaka, Zambia; Tropical Gastroenterology and Nutrition Group, University of Zambia, Lusaka, Zambia; Department of Medicine, University Teaching Hospital, Lusaka, Zambia; Department of Medicine, Levy Mwanawasa University Teaching Hospital, Lusaka, Zambia; Tropical Gastroenterology and Nutrition Group, University of Zambia, Lusaka, Zambia; Department of Research, Centre for Infectious Disease Research in Zambia, Lusaka, Zambia; Division of Gastroenterology, Massachusetts General Hospital, Boston, Massachusetts, USA; Division of Gastroenterology, Massachusetts General Hospital, Boston, Massachusetts, USA; Department of Infectious Diseases, Bern University Hospital, University of Bern, Bern, Switzerland; Institute of Social and Preventive Medicine, University of Bern, Bern, Switzerland; Division of Infectious Diseases, University of California Los Angeles, Los Angeles, California, USA; Tropical Gastroenterology and Nutrition Group, University of Zambia, Lusaka, Zambia; Blizard Institute, Queen Mary University of London, London, United Kingdom; Division of Gastroenterology, Massachusetts General Hospital, Boston, Massachusetts, USA; Department of Medicine, University Teaching Hospital, Lusaka, Zambia; Department of Research, Centre for Infectious Disease Research in Zambia, Lusaka, Zambia; Division of Infectious Diseases, University of Alabama Birmingham, Birmingham, Alabama, USA

**Keywords:** hepatitis B virus, Africa, liver fine-needle aspiration biopsy, single-cell RNA sequencing, human immunodeficiency virus

## Abstract

In Lusaka, Zambia, we introduced liver fine-needle aspiration biopsy (FNAB) into a research cohort of adults with treatment-naive chronic hepatitis B virus (HBV) infection, with and without human immunodeficiency virus (HIV) coinfection, as well as with acute HBV infection. From 117 enrollment and 47 longitudinal FNABs (at 1-year follow-up), we established participant acceptability and safety. We also demonstrated the quality of the material through single-cell RNA sequencing of selected enrollment FNAs, which revealed a range of immune cells. This approach can drive new insights into HBV immunology, informing cure strategies, and can improve our understanding of HBV natural history in Africa.

## Teaser

Liver fine-needle aspiration biopsy, analyzed via single-cell RNA sequencing, proved safe and acceptable, including when performed longitudinally, among participants in an HBV cohort in Zambia, offering a promising method to study HBV immunopathogenesis, including in low- and middle-income countries.

Major gaps persist in our knowledge of hepatitis B virus (HBV) immunopathogenesis [1] as limited studies have been performed at the site of infection—the human liver. Liver fine-needle aspiration biopsy (FNAB) [2] has potential to close knowledge gaps as it can reliably capture the immunodiversity within patients’ livers [3], and its lower potential for complications than core biopsy [2, 4] may make it more acceptable in longitudinal research studies. To date, early HBV research studies that included liver FNAB were conducted at top hepatitis research centers in North America and Europe; however, there is strong need to extend this type of HBV research to low- and middle-income countries. In sub-Saharan Africa, for example, there is a high burden of HBV infection, and unique host and viral factors that may alter the infection's natural history. In this article, we describe our introduction of liver FNAB, analyzed via single-cell sequencing, into a hepatitis B research cohort in Zambia (Southern Africa) to characterize the impact of human immunodeficiency virus (HIV) coinfection on HBV outcomes [5].

## METHODS

### HBV Cohort Description

We incorporated the liver FNAB procedure into a longstanding HBV cohort study in Lusaka, Zambia, which started in 2013 [6, 7]. The study has ethical approvals from the University of Zambia Biomedical Research Ethics Committee (Lusaka, Zambia) and the University of Alabama at Birmingham Institutional Review Board (Birmingham, AL). General inclusion criteria were 18 years of age and above, and seropositive for hepatitis B surface antigen. Participants fell into 3 groups: those with HIV coinfection and starting antiretroviral therapy for the first time; those who were HIV negative and eligible to start antiviral therapy for chronic HBV monoinfection; and those who were HIV negative and had acute HBV infection. Exclusion criteria for all groups were pregnancy, hepatitis C virus infection, clinical evidence of acute liver failure, or decompensated cirrhosis. All participants provided written informed consent prior to any protocol-specific procedures. Treatment for HBV monoinfection and HBV/HIV coinfection was per Zambian Ministry of Health guidelines. Liver FNABs were performed as part of enrollment and 1-year follow-up visits.

### Pre-FNA Safety Assessment

As soon as possible after enrollment or the 1-year visit, and within 1 week prior to the FNA procedure, participants underwent an abdominal ultrasound and blood tests, with results discussed during a weekly multidisciplinary call with research assistants who recruited the participants, nurses, hepatologists, and a laboratory technician. The FNA was canceled for ascites or liver lesions on ultrasound, international normalized ratio (INR) > 1.3, platelet count <80 000/mm^3^, or hemoglobin <8.0 g/dL. Those with concerning liver lesions on ultrasound were referred for a multiphase computed tomography scan to assess for hepatocellular carcinoma.

### FNA Collection, Processing, and Analysis

Ultrasound-guided percutaneous liver FNAB was performed by a Zambian hepatologist (E.S. and B.N.) with 4 passes collected from each participant at each time point. The liver FNAB sampling procedure is described in detail in the Supplementary Material.

At the local laboratory, FNAB passes underwent depletion of red blood cells by use of the EasySep RBC Depletion Reagent and the EasySep magnet (StemCell Technologies). After depletion, cells were counted and 15 000 to 20 000 cells were loaded into a HIVE cell loader (Honeycomb Biotechnologies) that was then frozen at −20°C; remaining cell aspirates were viably stored at −80°C in fetal bovine serum with 10% dimethyl sulfoxide. Frozen HIVEs were shipped to the analyzing laboratory within 6 months.

At the US laboratory, captured mRNA from HIVEs was amplified to create sequencing-ready libraries. Libraries were multiplexed and sequenced on the NovaSeq 6000 sequencer, using a NovaSeq SP 100-cycle flow-cell, and raw data (FASTQ files) were processed using BeeNet, a custom software to process paired-end Illumina sequencing data from libraries produced by the HIVE methodology. FASTQ files were quality controlled, preprocessed, aligned to Genome Reference Consortium Human Build 38 (GRCh38), and then transformed into annotated count matrices. Resulting count matrices were imported to Seurat for analysis and visualization. High-quality cells were selected for analyses using quality-control filtering with the following parameters that we established in a prior liver FNAB study: less than 40% mitochondrial RNA content, to account for the prevalence of highly metabolically active cells in the liver (eg, hepatocytes, macrophages, effector T cells) [8]; greater than 300 genes per cell; and >500 reads per cell [9]. After removing doublets, cells were clustered and differential gene expression analysis was performed using Seurat's built-in functions. Clusters were annotated according to canonical markers, significance defined as a Bonferroni-corrected *P* value less than .05 and a log_2_-fold-change of at least 0.25.

### Analysis of Participant Acceptability and Safety

We analyzed the numbers of participants who met safety criteria and returned for the procedure as planned (ie, not returning could indicate lack of acceptance). Following enrollment FNAB, we described losses to follow-up, which may have reflected dissatisfaction with the procedure. During a post-FNAB follow-up visit, we also conducted brief qualitative (open-ended) interviews with some participants to elicit their perspectives on the procedure. Interviews were based on a standard interview guide and were conducted by a staff member who did not participate in the procedure. Interviews were audio recorded, translated to English when necessary, and transcribed verbatim for analysis. To assess safety, we described any issues reported by participants just after the FNAB and at follow-up, and any complications.

## RESULTS

### Adaptation of the Liver FNAB Procedure for Zambia

**Figure 1.**
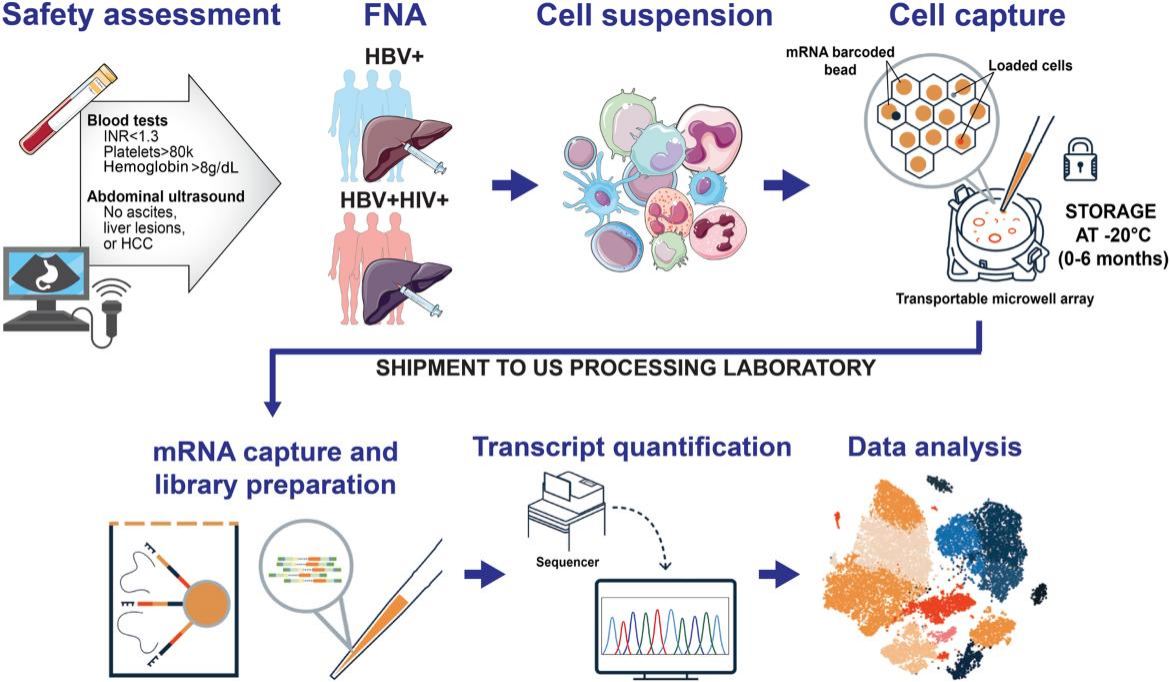
Overall summary of the liver FNAB study workflow. At the recruitment site in Zambia, following a safety assessment, liver aspirates were obtained and loaded onto transportable microwell arrays, which were shipped to the processing site in Boston for sequencing and analysis. Abbreviations: FNA, fine-needle aspiration; HBV, hepatitis B virus; HCC, hepatocellular carcinoma; HIV, human immunodeficiency virus; INR, international normalized ratio.

We held a series of virtual meetings to develop a standard operating procedure that leveraged US collaborators prior omics analysis of liver FNAs [9, 10], relied as much as possible on supplies and reagents that were locally available in Zambia, and took into consideration local health system and patient factors. We briefly used the original Seq-Well protocol, a platform with potential to make single-cell analysis possible with samples collected in low-resource settings. However, in 2020, we adopted its commercial version (HIVE scRNA solution), which requires much fewer and easier processing steps and allowed us to collect and process up to 4 FNABs at a time. A Zambian hepatologist (E.S. and B.N.), already experienced in liver core biopsy, and several Zambian laboratory technicians visited the US site to train in sample collection and processing. An immunologist-hepatologist from the US team visited the Zambia site to reinforce the training. During the initial period of implementation, a weekly virtual meeting was held to discuss each individual FNA procedure, and review the FNA appearance (ie, degree of visualized blood contamination), cell counts, and processing. The overall approach is summarized in Figure 1.

### Feasibility and Acceptability

From October 2020 to March 2023, we enrolled 157 participants, including 88 with HBV/HIV coinfection, 55 with chronic HBV monoinfection, and 14 with acute HBV infection (Supplementary Material). Median age was 34 years (range, 18–62 years) and 59 participants (37.6%) were women. Of 157 participants, 138 completed the pre-FNAB safety assessment; 125 of 138 (90.6%) met safety parameters, and 117 underwent the enrollment FNAB. Among the 13 failing safety screening, 9 had liver lesions, 3 of which were subsequently diagnosed as hepatocellular carcinoma. Among those completing the enrollment FNAB, we selected 64 participants for a repeat FNAB at their 1-year follow-up. Of them, 47 (73%) completed the second FNAB, with 5 (7.8%) declining the second procedure, 5 (7.8%) not meeting safety criteria, and 6 (9.4%) not having the FNAB due to staffing or supply issues. A participant flow chart is presented in Supplementary Figure 1.

We conducted qualitative interviews with 11 participants (3 with HIV and 2 with acute HBV infection) who underwent the FNAB, including 3 who had 2 FNABs (Supplementary Material). Short-lived pain at the puncture site was their main concern, and some additionally had periprocedure anxiety, especially before the first FNAB. However, pain resolved quickly and participants returned to their normal activities rapidly, even returning to work the same day in 1 case. Participants reported overall satisfaction with the study and their interactions with the study team, and this, plus transportation reimbursement available for study visits, made liver FNABs acceptable.

#### Patient Safety

Overall, there were no major complications in 164 FNAB procedures. In 1 participant, pain at the puncture site was so severe (puncture of the biliary tree was suspected as FNAB fluid was yellow), during and immediately postprocedure, that we admitted him to the hospital for 24 hours of clinical observation and pain management. We routinely phoned participants the day after FNAB, and on those calls <10% complained of ongoing pain at the puncture site. Only 5 participants returned to clinic to be reviewed by a doctor due to persistent pain. None of these required intervention beyond the prescription of paracetamol.

#### Quality and Usability of Liver FNAB Samples

**Figure 2.**
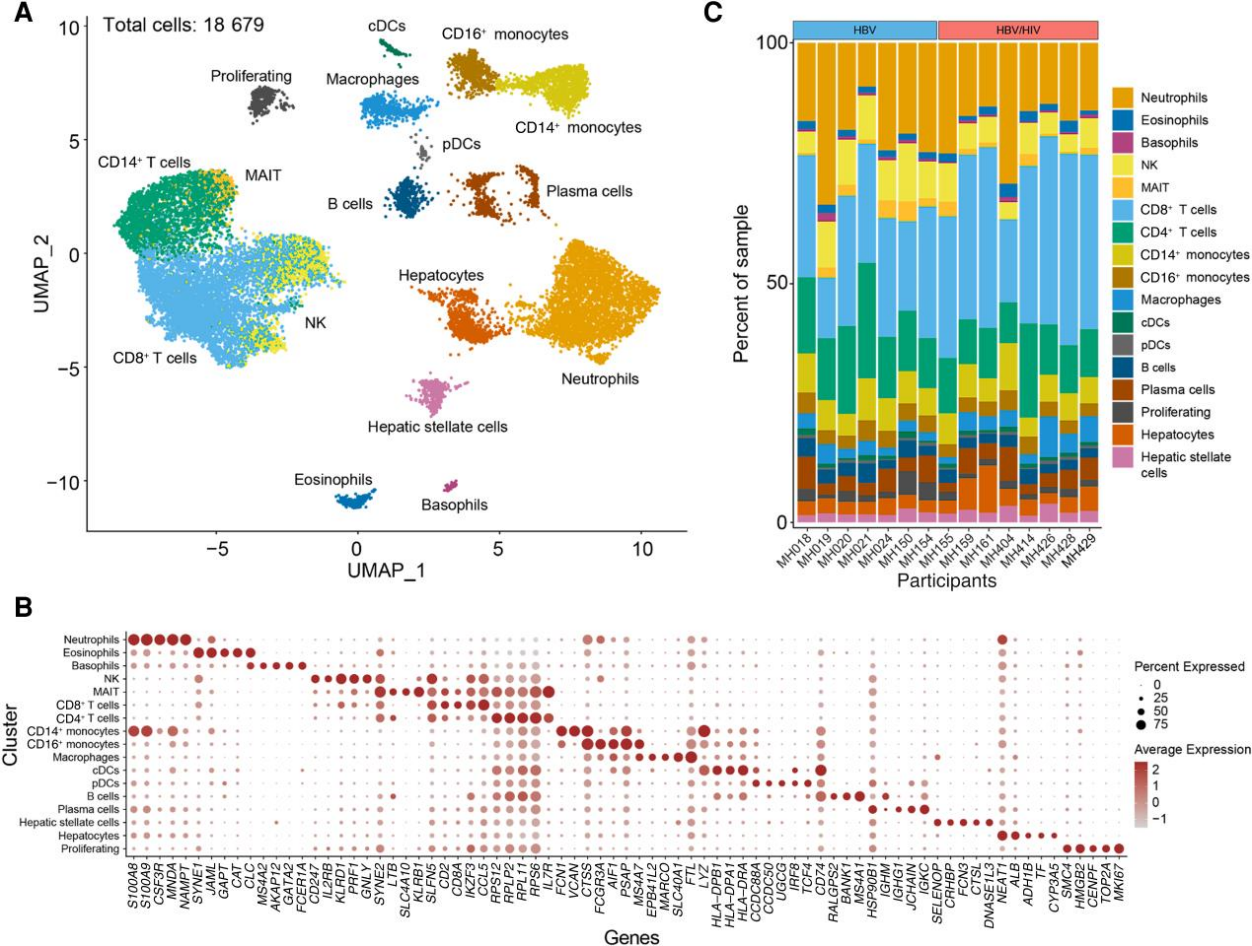
Display of the cell populations obtained from liver FNAB. *A*, UMAP of single cells annotated by cell type (cells included in analysis have >300 genes, > 500 UMIs, and <40 MT%). *B*, Dot plot showing marker gene expression for each identified cell type. *C*, Stacked bar plots grouped by infection status showing the proportion of identified cell types in each enrollment sample. Abbreviations: DC, dendritic cell; FNA, fine-needle aspiration; HBV, hepatitis B virus; HIV, human immunodeficiency virus; MAIT, mucosa-associated invariant T cell; MT, mitochondrial transcript; NK, natural killer cell; UMAP, uniform manifold approximation and projection; UMI, unique molecular identifier.

Cell counts were documented for 502 of 652 individual FNAB passes. Cells were present in 495 (98.6%) of the passes with a median number of 22 277 cells per FNAB pass (interquartile range, 15 000–32 375). So far, cDNA libraries were successfully created for 55 loaded HIVEs and 15 cDNA libraries from enrollment FNABs from people with chronic HBV with and without HIV were sequenced and analyzed. In line with the typical HIVE version 1 cell recovery of 20% at low thresholds (100 genes/200 transcripts per cell), we recovered approximately 3000 of the 15 000 loaded cells per participant FNAB (range, 9.40%–36.73%). After quality control filtering (Supplementary Figure 2*A* and 2*B*), and removal of doublets, we identified 18 679 high-quality cells (11 005 HBV/HIV coinfection; 7674 HBV monoinfection). We generated a uniform manifold approximation and projection (UMAP) to visualize the distribution of cells and performed clustering (Figure 2*A*). Clusters were identified based on significant differential expression of canonical marker genes (Figure 2*B*). We consistently captured major lymphoid and myeloid immune cell types in each patient, that is, T cells, B cells, neutrophils, monocytes dendritic cells, and macrophages alongside parenchymal cells such as hepatocytes, and hepatic stellate cells (Figure 2*C*).

## DISCUSSION

We report the use of liver FNAB in Zambia with subsequent molecular analysis of intrahepatic HBV immunity. FNAB was acceptable to many participants, including as a longitudinal procedure, and no major complications occurred in 164 initial procedures and 656 biopsy passes. This novel approach may open a new window into the pathogenesis of HBV in humans, including in low- and middle-income settings.

In Zambia, we documented high willingness of people with HBV monoinfection and HBV/HIV coinfection to participate in a study involving research liver biopsies, building on studies in high-income countries [10–12]. We readily recruited more than 150 participants and approximately 90% who met pre-FNAB safety criteria completed the procedure. Only approximately 10% of participants who were approached for a second FNAB declined it, indicating that despite frequent pain immediately after the FNAB, the procedure was perceived as acceptable. This was also reflected in qualitative interviews. We believe acceptability was driven by the local team's in-depth knowledge and experience in the management of HBV and their ability to explain the FNAB procedure to participants. However, we cannot fully exclude that some participants may have misconceived that there was a therapeutic benefit from the FNAB [13].

We also report that liver FNABs collected in Zambia yielded high-quality single-cell transcriptomic landscapes of the liver, comparable to studies conducted at top hepatitis research centers in upper-income countries [3, 9]. Optimization of sampling and processing required strong partnerships between experienced investigators in Boston and Lusaka, and there was a learning curve over a series of reciprocal training visits. Using the HIVEs also strongly facilitated data quality as fresh samples could be loaded in Zambia and then frozen for later analysis [9]. HIVEs also captured cell types that are not recovered well or at all after freezing, such as neutrophils, as well as small amounts of hepatocytes. Building on this success, we now hope to transfer additional liver FNAB processing and analysis steps to Zambia, to build African capacity for hepatitis research.

In conclusion, longitudinal liver FNABs, followed by molecular analysis at the single-cell level, were incorporated into an HBV research cohort in Zambia, to facilitate in-depth analysis of HBV liver immunology. This collaborative effort between liver-focused immunologists in the US and HBV clinical experts in Zambia is on track to reveal major insights into HBV immunopathogenesis in the context of the African endemic and may serve as a role model for similar advanced studies in low- and middle-income countries.

## Supplementary Data

Supplementary materials are available at *The Journal of Infectious Diseases* online (http://jid.oxfordjournals.org/). Supplementary materials consist of data provided by the author that are published to benefit the reader. The posted materials are not copyedited. The contents of all supplementary data are the sole responsibility of the authors. Questions or messages regarding errors should be addressed to the author.

## Supplementary Material

jiae054_Supplementary_Data

## Notes

***Financial support*.** This work was supported by the US National Institutes of Health (grant numbers U01AI069924, K01TW009998, R01AI147727, R01AI155140, R01AI148648, R01HD085862, and R37AI179640); and the Swiss National Science Foundation (grant number PP00P3_211025).
