# Supplementary material for: New Window Into Hepatitis B in Africa: Liver Sampling Combined With Single-Cell Omics Enables Deep and Longitudinal Assessment of Intrahepatic Immunity in Zambia
Source: J Infect Dis. 2024 Feb 9;230(5):e1171–5. doi: 10.1093/infdis/jiae054 (PMC11565906; doi:10.1093/infdis/jiae054)
Supplement: jiae054_Supplementary_Data [file jiae054_supplementary_data.docx]

**Supplementary data**

1. **Liver fine needle aspiration procedure description**

To initiate the FNA procedure following the initial examination by the hepatologist, a handheld ultrasound was used to identify and mark the puncture site. The puncture area was then cleansed and draped and using sterile technique, local anesthetic was injected in the skin, intercostal muscles, and liver capsule. subsequently, a sterile 25-gauge, 3.5-inch, Quincke-type, spinal needle was advanced along the anesthetized tract through the skin into the liver to a depth of 2-3 cm. The needle trocar was then removed and a 10ml syringe filled with 0.5ml of RPMI without phenol red was attached. Aspiration was done with gentle negative syringe pressure at the deepest extent of the needle pass, then aspiration was stopped as the needle was withdrawn from the liver so as to minimize hematic aspiration. Additional RPMI was drawn up in the syringe to ensure the entire aspirate had passed the needle and then the aspirate was placed in a 25ml falcon tube on ice. An additional 3 passes were made with the needle advanced in the same anesthetized tract but slightly different directions to avoid obtaining a bloody sample. A plaster was then placed at the puncture site and participants were monitored by a nurse with vital sign checks ¼ hourly for 1 hour before being released home. Changes in vital signs and/or significant pain prompted a review by the doctor. A nurse or research assistant phoned the patients the next day to check on well-being. FNAs were taken immediately to a laboratory within the same building for processing.

1. **Flow chart of the enrollment and retention of participants for liver FNA procedure.**


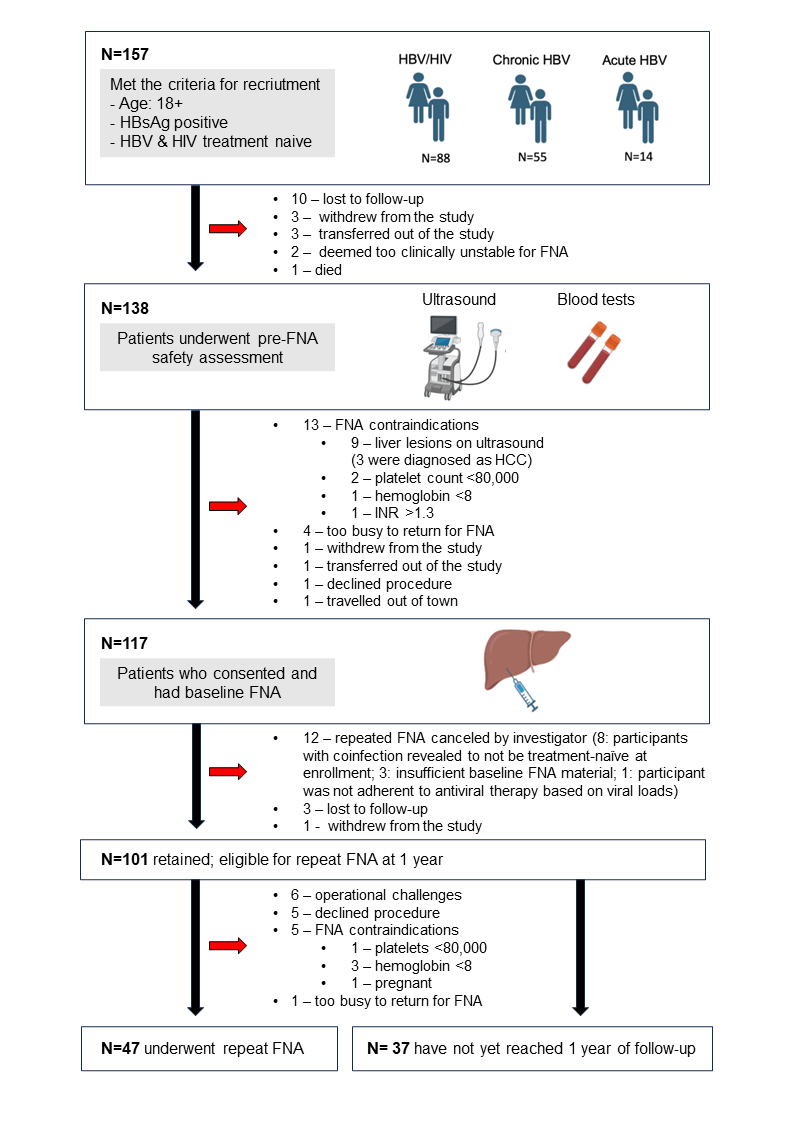


**Figure S1: Flow chart of enrollment and retention of participants for liver FNA procedure.** Black arrows indicate the patient participation process. Red arrows indicate patient dropout numbers and the corresponding reasons

1. **Graphs showing the quality of cells obtained from liver FNA**


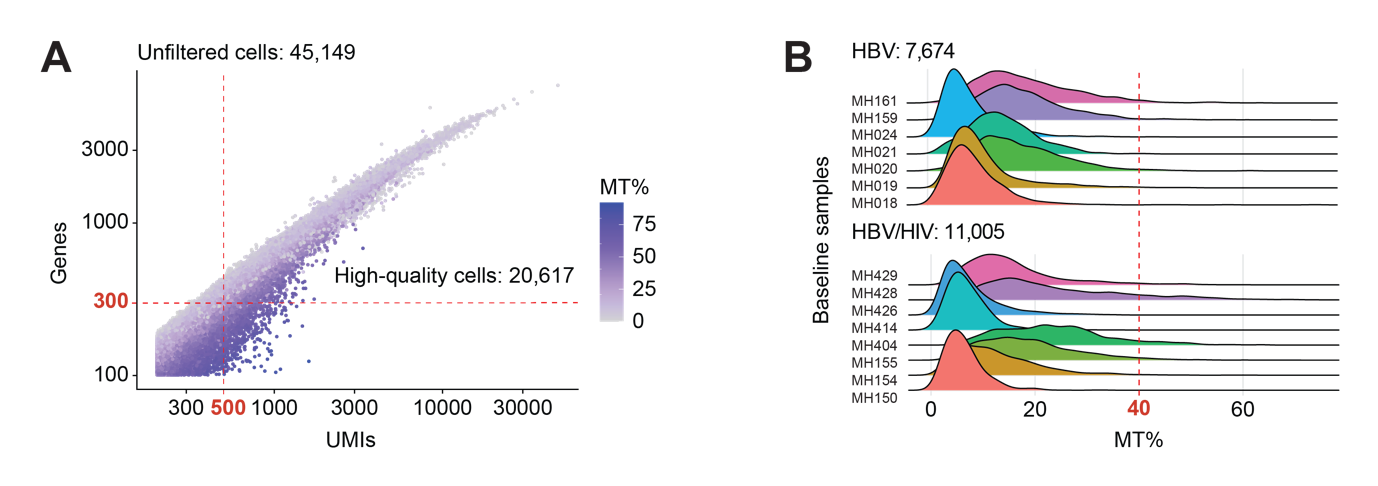


**Figure S2: Quality of cells obtained from liver FNA.** (A) Scatter plot showing the quality control metrics (genes, UMIs, and mitochondrial RNA fraction) of all sequenced cells from the 15 enrollment samples. High-quality cells were selected by identifying cells with >300 genes and >500 UMIs. (B) Ridge plots grouped by infection status showing each baseline sample’s distribution of high-quality cells by mitochondrial RNA content. Cells with <40 MT% were selected for further analysis.

1. **Qualitative interviews with participants who underwent liver FNA**

We conducted open-ended interviews with 11 participants who underwent at least one liver FNA. This included 3 with HBV/HIV coinfection, 6 with chronic HBV monoinfection, and 2 with AHB. Three interviewed participants underwent the FNA twice. Similar numbers of men and women were interviewed. Pain was far and away the main concern of participants regarding the procedure. Pain was most severe at the time of injection of local anesthetic; however, multiple patients also reported some pain during FNA passes. Most often the pain resolved shortly after the procedure; however, for some it lingered longer. A few quotations related to the pain of FNA are as follows:

*“…on the issue of the injection, I can say it is slightly painful, though it did not give me any problem; so, we can’t complain much because that’s how the procedure is and what we want is having life. Life is what is important, and so we just have to follow the procedure. Otherwise, we are so thankful for attending to us in a welcoming manner.” (32-year-old man with HBV/HIV coinfection)*

*“..the effect of the first injection for paralyzing or for causing numbness…I feel if they can be taking a bit of some time doing it so that the process of feeling the injection going in and out completely goes…I say so because at first, in as much as they did that, I still somehow felt the needle going in and out, and the like.” (*32-year-old male with chronic HBV monoinfection)

*“…and after the procedure was done, I think I just took a soft drink, and I was ok. I even went home then went back for work, and I was able to do my work throughout the day. I was very fine.”* (25-year-old man with HBV/HIV coinfection)

*“I was able to resume my normal activities right away just after the procedure. Then I didn’t have any problems removing the bandage, in that I was able to remove it in few hours. The other thing is that I just developed a little sore on the side I was injected, but it cleared away quickly.” (42-year-old man with chronic HBV monoinfection)*

*“I noticed that each time I wanted to lie in bed at night; I would feel some mild pain which went on for some days especially on the side where the procedure was done, but after some time the pain was gone. So as for now I am able to do all my routine works without any hindrances.” (*38-year-old woman with chronic HBV monoinfection)

For those who underwent liver FNA twice, experiences of pain were varied. A few quotations regarding repeat FNA are as follows:

*“…the second one, though I prepared myself, but I think the two injections were still very painful for me, then afterwards, my BP rose very high, and because of that I was told not to go immediately, saying they needed to observe me for some time. After some time, the BP normalized and I was then discharged. So, in short, the injections were so painful, and I think the pain is what triggered my BP to shot up, but then I just accepted everything and now am ok.”* (22-year-old woman with AHB)

*“The first one was so painful, and so I expected the same kind of pain for this second one even as I was coming, but surprising enough the pain today was not very severe as it were.”* (50-year-old man with HBV/HIV coinfection).
